# Supplementary material for: Coronavirus Pseudotypes for All Circulating Human Coronaviruses for Quantification of Cross-Neutralizing Antibody Responses
Source: Viruses. 2021 Aug 10;13(8):1579. doi: 10.3390/v13081579 (PMC8402765; doi:10.3390/v13081579)
Supplement: Supplementary file 1 [file viruses-13-01579-s001.zip › viruses-1236329-supplementary.pdf]

viruses

MDPI

Supplementary Materials

Figure S1 – HCoV neutralisation by human sera

Example neutralisation data by human sera is displayed in Figure 9 and described in the main text. Additional data from this panel of serum samples is shown below in **Supplementary Figure S1**. PVs were incubated for an hour at 37ºC with human sera prior to addition of target cells; neutralisation of entry was assayed as a reduction in luciferase output after 48 hours. The graphs shown below show the neutralisation curves from 5 SARS-CoV-2 seropositive samples and 5 SARS-CoV-2 seronegative samples against HCoV-HKU1, HCoV-229E and HCoV-NL63.

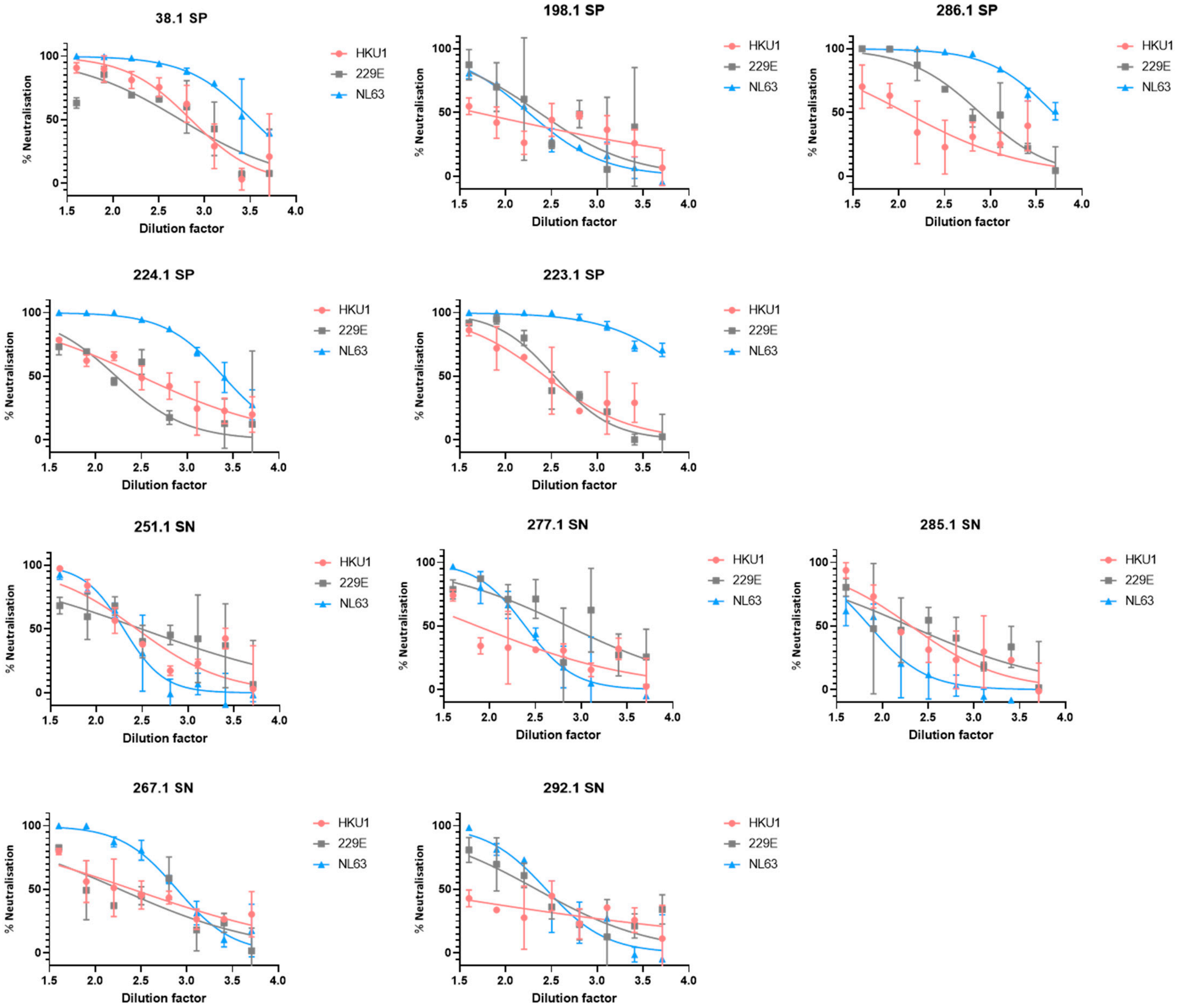

Figure S1. Neutralisation curves for 5 SARS-CoV-2 seropositive samples (38.1 SP, 198.1 SP, 286.1 SP, 224.1 SP and 223.1 SP) and 5 SARS-CoV-2 seronegative samples (251.1 SN, 277.1 SN, 285.1 SN, 267.1 SN and 292.1 SN) against HCoV-HKU1 (HKU1), HCoV-229E (229E), and HCoV-NL63 (NL63). Curves fit by non-linear regression using GraphPad Prism 9**.**

Table S1 – Statistical analysis

Further details on statistical tests used in analysis of datasets in Figures 4-7 are shown in **Supplementary Table S1**.

Table S1. Statistical analysis.

Figure	Statistical analysis
Figure 4a	Where datasets passed the normality test, a one way ANOVA test with Tukey's multiple comparison test was performed; otherwise, a Kruskal Wallis test with Dunn's multiple comparison test was used.
Figure 4b	A Kruskal Wallis test with Dunn's multiple comparison test was performed for this analysis
Figure 5b	A Kruskal Wallis test with Dunn's multiple comparison test was performed for this analysis
Figure 5c	A One Way ANOVA test with Tukey's multiple comparison test was performed for these datasets
Figure 7a	Where datasets had a normal distribution, an unpaired T-test was performed; otherwise, a Mann Whitney test was performed.
Figure 7b	A Mann Whitney test was performed on these datasets
